# A pilot study of a behavior-change intervention for preventive therapy among women at high risk for invasive breast cancer

**DOI:** 10.1186/s12911-026-03510-4

**Published:** 2026-04-18

**Authors:** Inimfon Jackson, Lisa M. Lowenstein, Sara Nofal, Parijatham S. Thomas, Therese Bevers, Viola Leal, Ana Nelson, Robert J. Volk, Abenaa M. Brewster

**Affiliations:** 1 Department of Breast Medical Oncology, Division of Cancer Medicine, The University of Texas MD Anderson Cancer Center, Houston, TX USA; 2 Department of Health Services Research, The University of Texas MD Anderson Cancer Center, Houston, TX USA; 3 Department of Clinical Cancer Prevention, The University of Texas MD Anderson Cancer Center, 1515 Holcombe Blvd, Unit 1360, Houston, TX 77030 USA; 4 Division of Cancer Medicine, Department of Breast Medical Oncology, The University of Texas MD Anderson Cancer Center, Houston, TX USA

**Keywords:** Behavior change, Intervention, Preventive therapy, Tamoxifen, Atypical hyperplasia, Lobular carcinoma in situ, High-risk women, Breast cancer

## Abstract

**Background:**

The use of preventive therapy in women at high risk for invasive breast cancer is associated with a significant reduction in breast cancer risk, however, uptake remains suboptimal outside of clinical trials. We conducted a pilot study of a behavior-change intervention to encourage high-risk women to take preventive therapy.

**Methods:**

Women aged 35–69 with a history of lobular carcinoma in situ (LCIS) or atypical hyperplasia (AH) receiving care at MD Anderson Cancer Center, Houston, Texas, were eligible. Following cognitive testing, the tool was field-tested using a pre-post design, comparing patients seen before its integration into clinical practice (pre-implementation cohort) with those seen after implementation (post-implementation cohort). Participants completed self-administered questionnaires assessing knowledge, treatment preferences, decisional conflict, shared decision-making and acceptability; clinicians completed surveys on their experiences with the tool. Descriptive analyses and standard tests of association were performed.

**Results:**

Of the 48 women who participated, 21 were in the post-implementation cohort. The majority of participants were White (75%) and non-Hispanic (81%), with a median age of 53 years. Most participants (90%) reported never needing help with reading health materials and were comfortable searching for health information online (81%). Women in the post-implementation cohort had higher knowledge about breast cancer preventive therapy compared to those in the pre-implementation cohort. However, only 38% correctly understood that side effects such as hot flashes and vaginal symptoms occur infrequently. After discussing with their clinician, 57% of women in the post-implementation cohort stated an intent to take preventive therapy compared to 70% in the pre-implementation cohort. Both groups reported low decisional conflict. The majority of women in the post-implementation cohort had positive perceptions of the tool. Furthermore, clinicians gave high ratings for the tool’s acceptability (median 4, IQR: 3.75–4.5), feasibility (median 4; IQR: 4-4.5) and appropriateness (median 4, IQR: 4-4.5), and found it somewhat/very helpful (100%) to patients with making decisions about preventive therapy.

**Conclusion:**

Our findings suggest that the behavior-change tool was associated with improved patient knowledge about preventive therapy and had good acceptability. Future research should focus on enhancing patient education about the likelihood and management of side effects, measuring treatment initiation and adherence and identifying effective strategies to integrate these tools into clinical practice to support informed decision-making.

**Supplementary Information:**

The online version contains supplementary material available at 10.1186/s12911-026-03510-4.

## Background

Breast cancer is the most common cancer with about 316,950 new cases of invasive breast cancer diagnosed in 2025. The widespread use of screening mammograms for breast cancer has contributed to an increase in the identification of both invasive breast cancer and premalignant lesions, such as, atypical hyperplasia (AH) and lobular carcinoma in situ (LCIS) [1, 2]. Though these premalignant lesions are associated with an increased risk of developing an invasive breast cancer, several randomized studies have reported a substantial reduction in invasive breast cancer risk among women with these lesions when treated with tamoxifen, raloxifene, and aromatase inhibitors compared to placebo [3–7]. Due to the strength of the scientific evidence, multiple organizations like the National Comprehensive Cancer Network (NCCN), U.S. Preventive Services Task Force (USPSTF), and American Society of Clinical Oncology (ASCO) recommend the use of preventive therapy for women with AH or LCIS [8–10]. However, the uptake of preventive therapy for breast cancer risk reduction remains suboptimal.

Studies have been conducted to understand the various factors that influence the uptake of breast cancer preventive therapy. Some of the key barriers identified include inadequate knowledge among both patients and clinicians, fear of side effects, concerns about similarity to chemotherapy, and poor communication between patients and their providers [11–15]. Additionally, adherence to these medications is suboptimal due to concerns about side effects, lack of knowledge among patients and providers and insufficient time for counseling [13, 15–17]. A systematic review that evaluated the uptake and adherence to preventive therapy for women at increased risk for breast cancer, reported the uptake estimate at 16.3% [15]. Preventive therapy uptake was significantly higher among clinical trial patients than in non-trial patients (25.2% vs. 8.7%, *p* < 0.001) [15]. We previously showed that a systemic level intervention administered at a single institution and focused on women with a newly diagnosed or existing premalignant lesion could increase the prescribing of preventive therapy to 82% and 48%, respectively [18]. The primary patient reported reasons for not choosing to start preventive therapy were the potential medication side-effects and a disbelief of the increased risk of breast cancer. There is a need to provide women with premalignant lesions for whom preventive therapy is strongly recommended, a better understanding of their risk for developing an invasive breast cancer and a strong rationale for the benefits of preventive therapy [10, 15]. 

Decision support tools have been shown to improve communication between patients and their healthcare providers [19–21] and may facilitate the discussion about preventive therapy. While some studies have reported that decision tools can increase the knowledge about preventive therapy, their impact on preventive therapy initiation is unclear [22–24]. For instance, Kukafka et al. conducted a pilot study using a decision support tool for breast cancer preventive therapy among high-risk women and found that while it led to a better perception of their breast cancer risk, none decided to initiate preventive therapy [22]. Furthermore, there was an increase in decisional conflict post-exposure to the decision support tool [22]. Using decision support principles to structure information presentation, we developed a guideline-concordant behavior-change intervention for use in the clinical setting to nudge or encourage preventive therapy use among women with AH and LCIS given that clinical guidelines recommend treatment [25]. Blumenthal-Barby et al., has argued that “nudging” a patient toward specific options when using patient decision aids can be ethically justified over more balanced, nondirective approach [26]. The key components of the tool included a patient-centric educational video and visual representation of the potential benefits of preventive therapy. Our primary objective was to evaluate whether implementation of the tool was associated with increased intent to take preventive therapy. We compared outcomes between participants who received usual care and those who were exposed to the intervention.

## Methods

### Study population

Women aged 35 to 69 years newly diagnosed with LCIS or AH and receiving care in the Cancer Prevention Center at the MD Anderson Cancer Center, Houston, Texas, were eligible to participate in this study. Exclusion criteria included: (1) non-English speaking, (2) previous completion of 5 years of preventive therapy and (3) premenopausal status with a personal history of blood clots or stroke. Since it is unclear that the benefits of preventive therapy outweigh the risks for women over age 70, this population was excluded from the study. Clinicians credentialed to see patients at MD Anderson for the management of high-risk breast lesions were also invited to participate. The study was reviewed and approved by the MD Anderson Cancer Center Institutional Review Board (IRB), and all study participants provided written informed consent.

## Study interventions

### Design of the behavior-change tool

While we used decision support principles to structure information presentation, the goal of the tool was to promote intent to take preventive therapy in women at high-risk of invasive breast cancer due to a diagnosis of LCIS or AH. The tool was developed by the research team consisting of medical content experts (AMB and TB), decision science experts (RJV and LML), and a patient advocate, with components of the tool produced by MD Anderson staff. The tool used constructs from the Integrative Model of behavior to target knowledge, perceived risk, attitudes, normative pressure, and self-efficacy to promote breast cancer chemoprevention [27, 28]. 

**Fig. 1:**
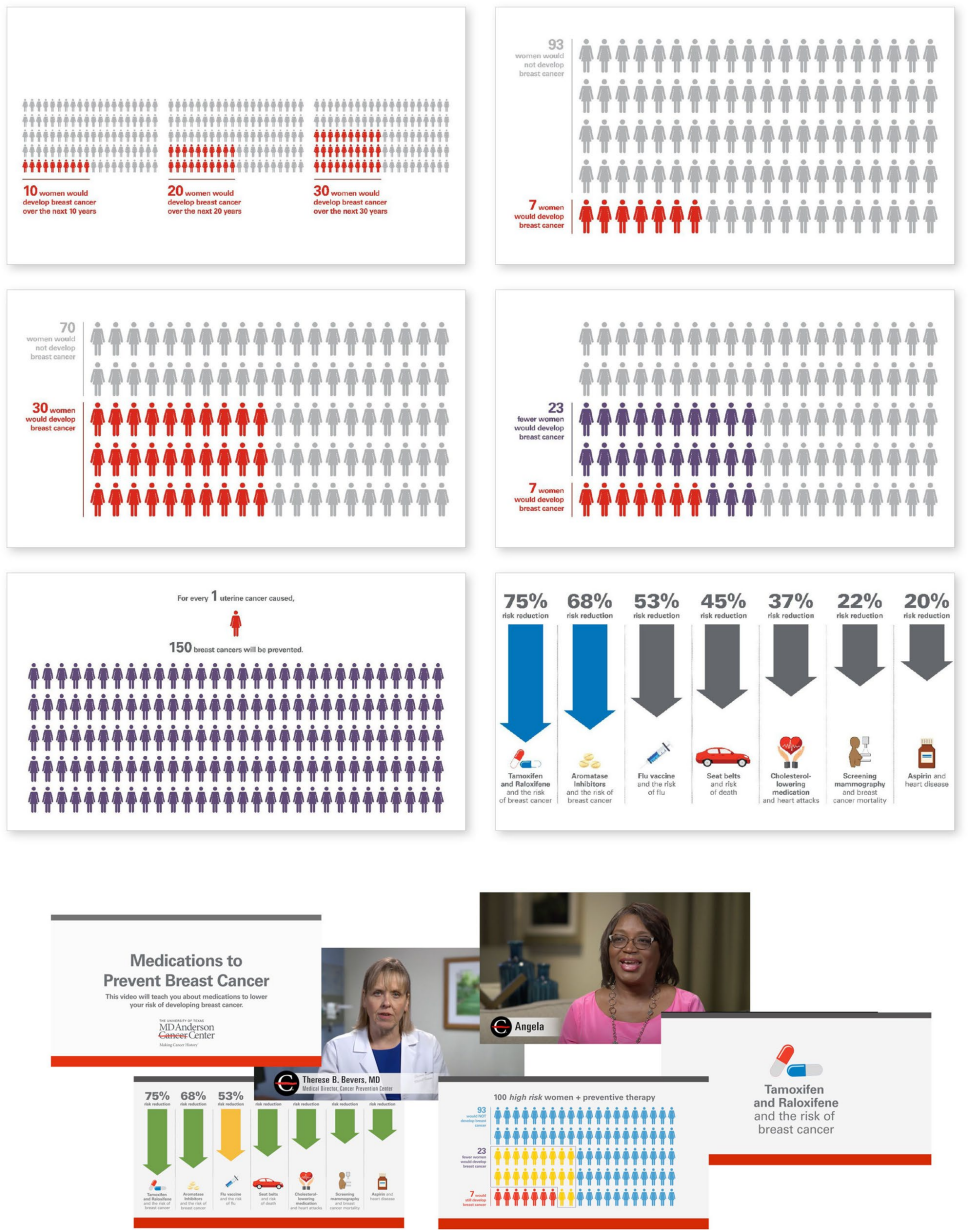
Screenshots of the behavior-change tool, including components from the educational video and graphic displays reviewed by patients

The behavior-change intervention included two components: (1) an educational video about the preventive therapy for review by women outside of the clinical encounter, including its benefits and possible side-effects, and (2) web-based graphic displays of the magnitude of breast cancer risk reduction with preventive therapy for review during the clinical encounter. The video (4:52 in length) included (a) a narrated overview of preventive therapy, (b) information about the magnitude of benefit compared to other prevention strategies, such as flu vaccinations and cholesterol lowering medications to prevent cardiovascular outcomes, and (c) the magnitude of benefit in reducing breast cancer incidence from preventive therapy in high-risk women. A unique feature of the video were testimonials from three women who had taken preventive therapy, describing reasons why they initiated preventive therapy and their experience with side effects. The second feature was a series of icon arrays for four age groups of women (30–39 years, 40–49 years, 50–59 years, and 60–69 years), showing the risk of developing breast cancer over the next 30 years without and with preventive therapy. These visual depictions of risk were meant to be viewed during the clinical encounter when the decision about preventive therapy is discussed (see Fig. 1 for screenshots from the intervention).

## Cognitive testing

Patients seen at MD Anderson who met eligibility criteria were recruited to participate in iterative rounds of cognitive testing of the tool that consisted of (1) an educational video and (2) visual representation of the benefits of preventive therapy and the risk reduction with treatment. Participants reviewed drafts of tool for clarity as part of a cognitive interviewing process. For each round of cognitive testing, a trained study coordinator facilitated the process by asking patients to think aloud while reviewing the materials, documenting their feedback throughout the interview. Interviews were conducted in person and lasted about one hour. Patients were asked to restate the content shown in their own words; were asked to summarize what each visual representation of benefit and risk was meant to show; were asked what parts were difficult to understand, and why; were probed on their understanding of specific words or abbreviations such as “AH/LCIS;” and were asked if they related to the women in the testimonials.

## Study design and field testing

We conducted a sequential pre-post study design of the tool [29]. Patient participants were categorized into two time-based cohorts: (1) patients seen before the tool was implemented (pre-implementation [usual care] cohort) and (2) patients seen after its incorporation in clinical practice (post-implementation [intervention] cohort). The pre-implementation phase was conducted from July 2019 to March 2020 while the post-implementation phase took place from August 2021 to April 2023. Field testing was performed to compare outcomes for participants and their clinicians in the pre-implementation phase with those in the post-implementation phase. The pre-implementation cohort received the usual care with clinicians discussing and recommending preventive therapy during the clinical consultation as per clinical guidelines [25]. Those in the post-implementation cohort viewed the educational video in the waiting room or exam room immediately before meeting with their clinician in the clinic. The visual representation of the benefits of preventive therapy and risk reduction with treatment was used by the clinician during their clinical consultation with the patient. Enrolled patients in the pre-implementation and post-implementation cohorts were given questionnaires to complete in person or via email after their clinical encounter.

### Outcome measures and data collection

Patients with newly diagnosed with AH or LCIS were identified in the undiagnosed breast clinic at MD Anderson. They were contacted via phone or at their clinic visit and invited to participate in the study. All patients seen in the undiagnosed breast clinic were approached for their interest in the study and those who consented were enrolled in the study. Study participants completed questionnaires that were administered electronically via REDCap (Research Electronic Date Capture) (which has confidentiality as one of its core design principles) for sociodemographic information, knowledge about preventive therapy, leaning scale, intent to take preventive therapy after clinician consultation, decisional conflict [30], shared decision-making process [31] and Ottawa acceptability scales. Sociodemographic data included age, race, ethnicity, education level, insurance type, relationship status and religion. Health literacy was assessed through questions regarding the frequency of needing assistance with reading [32], and access to computers, smartphones, and the internet. Additional questions were included to gauge participants’ healthcare decision-making preferences.

The primary outcome was participants’ intent to take preventive therapy following their clinician consultation. This was assessed using a survey question that all participants completed indicating whether their intent was to take preventive therapy, not take preventive therapy or if they were unsure.

Knowledge about preventive therapy was assessed with 6 multiple choice items that were developed by the study team with feedback from clinicians and patients who participated in the cognitive testing of the tool. Wording was modified to ensure that the items were clear and understandable.

The Ottawa measure of decision/choice disposition was utilized to explore leaning preferences in both pre-implementation and post-implementation cohorts. The 15-point choice predisposition scale was scored and interpreted as: 1–5 (yes); 6–10 (unsure); or 11–15 (no) [33]. It has a test-retest Pearson correlation coefficient greater than 0.90 [30]. 

Decisional conflict was measured using the 16-item Decisional Conflict Scale. The 16-item Decisional Conflict Scale is a validated patient-reported measure that assesses personal uncertainty about which course of action to take when choosing among competing health care choices. It is composed of 16 items rated on a Likert scale from strongly agree to strongly disagree. Scores range from 0 to 100 with high scores indicating more decision uncertainty. The Cronbach’s alpha ranges from 0.78 to 0.97 [27]. 

The 4-item version of the Shared Decision-making Process survey was used to assess patient involvement in the decision-making process. It is a brief 4-item patient-reported measure that assesses the amount of shared decision making occurring around a medical decision. It generates scores from 0 to 100, where higher scores indicate greater shared decision making. The Cronbach’s alpha ranges from 0.78 to 0.97 [28]. 

Participants in the post-implementation cohort completed the Ottawa acceptability questionnaire to evaluate the comprehensibility and suitability of the tool [34]. 

Clinicians who provided care to the patients in the post-implementation (intervention) cohort completed a questionnaire to assess whether the tool was useful, how much information was used, how patients reacted to the information provided, what impact the tool had on patients’ decisions, and to identify any problems with the tool that could be addressed. Clinician survey data also included the 12-item survey to assess the Acceptability of Intervention Measure (AIM), Intervention Appropriateness Measure (IAM), and Feasibility of Intervention Measure (FIM). These are three brief validated implementation outcome measures to assess distinct aspects of how healthcare providers perceive interventions. Each measure consists of 4 items rated on a 5-point Likert scale. It is calculated by summing the scores of different scales and dividing by four, with scores of four or more deemed the intervention to be acceptable, feasible, and/or appropriate. The Cronbach’s alpha 0.85 to 0.91) [35]. 

### Statistical analysis

Sociodemographic and clinical characteristics, knowledge about preventive therapy, and physician questionnaires were summarized by group. Preventive therapy knowledge was assessed by calculating the percentage of total correct responses within each intervention group. The Decisional Conflict Scale and subscale scores, shared decision-making process scores, and Ottawa measure of decision predisposition scores were calculated by group, with medians and inter-quartile ranges (IQRs) reported. Non-parametric tests were used due to the small sample size. Specifically, Fisher’s exact test and Wilcoxon Rank Sum test were used to compare categorical and continuous outcomes, respectively, between the pre-implementation and post-implementation cohorts. Effect sizes were estimated using the Hodges–Lehmann approach, representing the median difference between groups, along with corresponding 95% confidence intervals (CIs). These estimates provide a measure of the magnitude and direction of group differences and were emphasized over statistical significance given the exploratory nature of the study. All analyses were conducted using Stata SE version 16.1 (StataCorp, College Station, TX), with a p-value < 0.05 considered statistically significant.

## Results

### Cognitive testing

Of the seven women approached to take part in cognitive testing, four enrolled and completed the cognitive interview. All were ages 53–65 with private health insurance or TriCare benefits. All were non-Hispanic, three reported white race and one declined to report her race. One reported a high school diploma or GED, two had a college degree, and one had an advanced degree.

Based on feedback from cognitive testing, the draft content was well-understood. For example, in the visual display comparing the benefit of risk prevention behaviors, the patients understood that tamoxifen and raloxifene are the most effective at prevention compared to other measures. One mentioned surprise at the magnitude of the benefit compared to other types. All four patients understood what was meant by “average risk.” Three of the four patients understood that there was a low risk of uterine cancer. One patient noted that she was previously unaware that preventive therapy could cause uterine cancer.

Some feedback was not actionable in the context of this study. For example, one patient asked for more information on other ways to reduce breast cancer risk. Another patient wanted to know all factors (besides LCIS and AH) that could increase a woman’s risk for breast cancer. Two of the women noted that the patient stories did not match their personal experience with side effects.

### Participant sociodemographic characteristics (field test)

Of the 88 women who were approached to participate in the field test, 48 women enrolled in and completed the study, including 21 in the post-implementation cohort. There were no statistically significant differences in sociodemographic characteristics between groups. Most of the participants were White (75%), followed by Asian (15%), and approximately 81% identified as non-Hispanic or Latino. The median age was 53 years (IQR: 46–62), and most participants (69%) were married or in a relationship. About 32% had a graduate degree, 28% had a bachelor’s degree, and another 28% had some college education without a degree or an associate’s degree. Most women (92%) reported having private health insurance (e.g., BlueCross BlueShield, Aetna, Humana, UniCare), 13% had Medicare or Medicaid, and 4% reported TriCare or VA benefits (Table 1).

**Table 1:** Participants’ Sociodemographic Characteristics (*N* = 48)

Age, median (IQR)	Pre-implementation (*n* = 27)	Post-implementation (*n* = 21)	Total (*N* = 48)	*p*-value
	53 (45–62)	53 (47–58)	53 (46–62)	1.00
Race*				
White	20 (74.07%)	16 (76.19%)	36 (75.00%)	1.00
Black or African American	2 (7.41%)	1 (4.76%)	3 (6.25%)	1.00
Asian	4 (14.81%)	3 (14.29%)	7 (14.58%)	1.00
Other	1 (3.70%)	1 (4.76%)	2 (4.17%)	1.00
Ethnicity				1.00
Hispanic or Latino	5 (19.23%)	4 (19.05%)	9 (19.15%)	
Non Hispanic or Latino	21 (80.77%)	17 (80.95%)	38 (80.85%)	
Education^a^				0.080
Less than High school	0 (0%)	0 (0%)	0 (0%)	
High school grades 9–12, no degree	2 (7.69%)	2 (9.52%)	4 (8.51%)	
High school graduate or equivalent	0 (0.00%)	2 (9.52%)	2 (4.26%)	
Some college/Associate’s degree	11 (42.31%)	2 (9.52%)	13 (27.66%)	
Bachelor’s degree	6 (23.08%)	7 (33.33%)	13 (27.66%)	
Graduate degree	7 (26.92%)	8 (38.10%)	15 (31.91%)	
Health Insurance*				
Private insurance (for example, BlueCross BlueShield, Aetna, Humana, UniCare)	23 (82.14%)	21 (87.50%)	44 (91.67%)	0.71
Medicare or Medicaid	4 (14.29%)	2 (8.33%)	6 (12.50%)	0.67
Goldcard/Harris Health System coverage	0 (0%)	0 (0%)	0 (0%)	
TriCare or VA benefits	2 (7.14%)	0 (0.00%)	2 (4.17%)	0.49
Marital Status				1.00
Single/Divorced/Widowed	8 (29.63%)	7 (33.33%)	15 (31.25%)	
Married/In a relationship	19 (70.37%)	14 (66.67%)	33 (68.75%)	

IQR: Inter-quartile rangeP-value for age is based on Wilcoxon Rank Sum test. For all other variables, p-value is based on Exact Fischer test*For race and health insurance, respondents could select more than one option. Percentages and p-values represent those who selected vs. did not select each option of these variables.^a^ Some college (no degree); Associate’s degree (occupational or academic degrees); Bachelor’s degree (BA, BS, AB, etc…); and Graduate degree included master’s degree (MA, MS, MSW, etc…), professional school degree (MD, DDC, JD, etc…), or doctoral degree (PhD, EdD, etc…)

### Health literacy, technology access, and decision-making preferences (field test)

There were no statistically significant differences between groups in terms of health literacy and technology access. Most participants (90%) reported never needing help when reading instructions, pamphlets, or other written materials from their doctor or pharmacy. All participants used smart phones, and 98% had regular access to a computer and internet at home. The majority were comfortable sending and receiving text messages (96%), emails (92%), using the internet (85%), and searching for healthcare information online (81%) **(**Table 2**)**.

**Table 2:** Decision making preferences, access to and comfort with technology, and health literacy

	Pre-implementation (*n* = 27	Post-implementation (*n* = 21)	Total (*N* = 48)	*p*-value	
When making decisions about your healthcare, how much information do you prefer?				0.35	
The key facts about each of the treatment options	3 (12.00%)	2 (10.00%)	5 (11.11%)		
The key facts, plus some detail about the options I am interested in	10 (40.00%)	4 (20.00%)	14 (31.11%)		
The key facts and lots of details about all of the options	12 (48.00%)	14 (70.00%)	26 (57.78%)		
When making decisions about your healthcare, which of the following do you prefer?				0.60	
I prefer to hear about my treatment options from my doctor(s), then make the decision myself	4 (14.81%)	3 (14.29%)	7 (14.58%)		
I prefer to discuss my treatment options and my preferences with my doctor(s), then we make the decision together	21 (77.78%)	18 (85.71%)	39 (81.25%)		
I prefer to discuss my treatment options and my preferences with my doctor(s), then my doctor(s) should make the final decision	2 (7.41%)	0 (0.00%)	2 (4.17%)		
How often do you need to have someone help you when you read instructions, pamphlets, or other written material from your doctor or pharmacy?				0.38	
Never	23 (85.19%)	20 (95.24%)	43 (89.58%)		
Rarely	3 (11.11%)	0 (0.00%)	3 (6.25%)		
Sometimes	1 (3.70%)	1 (4.76%)	2 (4.17%)		
Often	0 (0.00%)	0 (0.00%)	0 (0.00%)		
Always	0 (0.00%)	0 (0.00%)	0 (0.00%)		
Do you have regular access to a computer and Internet access at home?				1.00	
Yes	26 (96.30%)	21 (100.00%)	47 (97.92%)		
No	1 (3.70%)	0 (0.00%)	1 (2.08%)		
Do you use a smartphone?					
Yes	27 (100.00%)	21 (100.00%)	48 (100.00%)		
No	0 (0.00%)	0 (0.00%)	0 (0.00%)		
Which of the following are you comfortable doing? *					
Sending and receiving text messages	25 (89.29%)	21 (87.50%)	46 (95.83%)	1.00	
Sending and receiving emails	23 (82.14%)	21 (87.50%)	44 (91.67%)	0.71	
Using the Internet	21 (75.00%)	20 (83.33%)	41 (85.42%)	0.52	
Searching for healthcare information online	19 (67.86%)	20 (83.33%)	39 (81.25%)	0.34	
Watching videos online	19 (67.86%)	19 (79.17%)	38 (79.17%)	0.53	
Filling out forms online	19 (67.86%)	20 (83.33%)	39 (81.25%)	0.34	

P-values are based on Exact Fischer test*For comfort with technology, percentages and p-values represent those who selected vs. did not select each statement

Similarly, there was no statistically significant difference between groups in terms of their decision-making preferences. Approximately 58% of women preferred receiving information that included both key facts and detailed explanations of all available options, 31% preferred receiving key facts with additional detail only about the options they were interested in, and just 11% preferred information limited to key facts for each treatment option. Regarding decision-making preferences, 81% preferred to discuss treatment options and personal preferences with their doctors(s) and make the decision together. A smaller proportion (15%) preferred to hear about the options from their doctor(s) and then make the decision themselves, while only 4% preferred to have their doctor(s) make the final decision after discussing the options and their preferences (Table 2).

### Participant knowledge of breast cancer preventive therapy (field test)

Women in the post-implementation cohort had greater knowledge of breast cancer preventive therapy compared to those in the pre-implementation cohort. All participants in the post-implementation cohort correctly identified that women with LCIS or AH are at higher risk of developing breast cancer than women without these conditions. About 95% of all women correctly recognized that preventive therapy can reduce breast cancer risk by up to 75% and that it is most effective when taken for five years. While all the participants were aware that preventive therapy may cause side effects such as hot flashes and vaginal symptoms, only 38% in the post-implementation cohort correctly understood that such side effects occur infrequently. Finally, 90% of all women correctly identified that preventive therapy is more effective at reducing breast cancer risk than other preventive health measures, such as taking cholesterol-reducing medication to prevent a heart attack or getting a flu shot to prevent influenza (Supplementary Table 1).

### Decisional conflict and shared decision-making process (field test)

Women in the post-implementation cohort reported low decisional conflict, with a total median score of 14.06 (IQR: 3.13-25) on the Decisional Conflict Scale. Among the Decisional Conflict subscales, the median scores were similar between groups (Table 3).

**Table 3:** Results of Decisional Conflict Scale and Shared Decision-Making Process Scale Scores

	Pre-implementation (*n* = 27)	Post-implementation (*n* = 21)	*p*-value	HL (95 CI%)
	Median(IQR)	Median(IQR)		
Decisional Conflict Scale Total Score	9.38 (0–25)	14.06 (3.13-25)	0.50	1.56 (-3.13, 12.50)
Informed Subscale	0 (0–25)	0 (0–25)	0.53	1.54 (-2.18, 8.33)
Values Clarity Subscale	0 (0–25)	0 (0–25)	0.83	2.67 (-4.31, 5.90)
Support Subscale	0 (0-16.67)	0 (0–25)	0.30	5.19 (-4.79, 8.33)
Uncertainty Subscale	16.67 (0-33.33)	25 (0-41.67)	0.40	3.63 (-1.37, 2.50)
Effective Decision Subscale	18.75 (0-31.25)	25 (0-31.25)	0.62	9.28 (-6.25, 6.25)
Shared Decision-Making Process Scale Score	4 (3–4)	4 (3–4)	0.16	5.85 (-8.50, 9.99)

IQR: Inter-quartile range

HL: Hodges–Lehmann estimated differences between groups

P-values are based on Wilcoxon Rank Sum test

The median scores on the Shared Decision-Making Process Scale were similar among participants in the post-implementation and pre-implementation cohorts. Overall, both groups reported high scores, indicating a good quality shared decision-making process **(**Table 3**)**.

### Ottawa measure of decision predisposition and preference (field test)

There were no statistically significant differences between groups on the Ottawa measure of decision predisposition (*p* = 0.6) or women’s preference and decisions regarding preventive therapy (*p* = 0.43). Women in the post-implementation cohort leaned toward taking preventive therapy, with a median score of 13 (IQR: 8–15) on the Ottawa measure of decision predisposition. After speaking with their clinicians, 57% of women reported an intent to take preventive therapy, 29% remained uncertain, and 14% chose not to take preventive therapy. Among those who were unsure, reasons included wanting to explore other preventive options or concerns that existing health problems might make preventive therapy unsuitable. Those who declined preventive therapy cited reasons, such as concerns about side effects and comorbidities, preserving quality of life, and family-related considerations. Similarly, women in the pre-implementation cohort leaned toward taking preventive therapy, with a median score of 15 (IQR: 8–15). After clinician consultation, 70% of participants reported an intent to take preventive therapy, 26% were unsure, and 4% decided against it. Uncertainty in the pre-implementation cohort was mainly driven by concerns about side effects, a preference for continued screening, or waiting on final pathology results before making a decision.

### Post-implementation cohort acceptability of the intervention measure (field test)

Most women in the post-implementation cohort had positive perceptions of the tool. 90% found the tool useful when making decisions about preventive therapy, and 75% felt it provided enough information to guide decision-making. All participants thought it was easy to identify their risk level using the tool, and 89.5% felt that the video component made the decision easier. Most participants believed the length (90%) and amount of information (80%) provided were appropriate. 75% of participants perceived the materials as slanted towards taking preventive therapy. When evaluating the presentation of information within the tool, most participants rated the potential benefits of preventive therapy (95%), potential harms (60%), risk of developing breast cancer (90%), the effectiveness of preventive therapy (89.5%), and the included patient stories (84%) as good or excellent (Table 4).

**Table 4:** Participants’ acceptability of preventive therapy in the implementation group (*n* = 21)

	*n*(%)
The potential benefits of preventive therapy	
Poor/Fair	1 (5.00%)
Good/Excellent	19 (95.00%)
The potential harms of preventive therapy	
Poor/Fair	8 (40.00%)
Good/Excellent	12 (60.00%)
My risk of developing breast cancer	
Poor/Fair	2 (10.00%)
Good/Excellent	18 (90.00%)
The effectiveness of preventive therapy	
Poor/Fair	2 (10.53%)
Good/Excellent	17 (89.47%)
Stories from women on preventive therapy	
Poor/Fair	3 (15.79%)
Good/Excellent	16 (84.21%)
The length of the materials was	
Too long	0 (0.00%)
Too short	2 (10.00%)
Just right	18 (90.00%)
The amount of information was	
Too much information	0 (0.00%)
Too little information	4 (20.00%)
Just right	16 (80.00%)
I found the materials	
Slanted toward taking preventive therapy	15 (75.00%)
Slanted toward not taking preventive therapy	0 (0.00%)
Balanced	5 (25.00%)
Would you have found this decision aid useful when you were making your decision about preventive therapy?	
Yes	18 (90.00%)
No	2 (10.00%)
What did you think of the way the tool showed your risk of developing breast cancer? Was it…	
Easy to find your risk level, or	20 (100.00%)
Difficult	0 (0.00%)
What did you think of the video? Did it make the decision…	
Easier	17 (89.47%)
More difficult	2 (10.53%)
Did you think we included enough information to help a woman make a decision about preventive therapy?	
Yes	15 (75.00%)
No	5 (25.00%)

### Providers’ perceptions of the intervention measure (field test)

Clinicians (three physicians and nine Advanced Nurse Practitioners) rated the tool highly across all implementation outcomes. On the AIM, IAM, and FIM scales, where scores range from 1 (completely disagree) to 5 (completely agree), providers rated the tool as acceptable (median: 4, IQR: 3.75–4.5), appropriate (median: 4, IQR: 4-4.5), and feasible (median: 4, IQR: 4-4.5). The tool also received a high usability rating on the System Usability Scale, with a median of 73.75 (IQR: 65–85). On the satisfaction scale (1 = very unsatisfied to 9 = very satisfied), providers reported high satisfaction (median: 8, IQR: 7-8.5). All clinicians either agreed (58%) or strongly agreed (42%) that the tool helped patients better understand the benefits and harms of preventive therapy. Most clinicians agreed (42%) or strongly agreed (33%) that it encouraged patients to spend more time considering the tradeoffs involved. 33% of clinicians reported that the tool made the visit somewhat longer, while 75% reported that the tool improved the quality of conversations with their patients. Most clinicians (83%) believed the amount of information provided was “just right”. While 55% of clinicians felt the tool was balanced, 45% believed the tool favored taking preventive therapy. 67% of clinicians said the time needed to review information in the tool was appropriate. All clinicians found the tool “somewhat” (67%) or “very” (33%) helpful to patients in making a decision about preventive therapy. Most clinicians believed that the tool had either “some” (64%) or a “great” (18%) impact on patients’ choices regarding preventive therapy. Lastly, 90% of clinicians said they would use the tool again, and 80% said they would recommend the tool to their colleagues (Table 5).

**Table 5:** Clinical providers’ perceptions of acceptability, appropriateness, and feasibility of the intervention measure (*N* = 12)

	median(IQR)
System Usability Scale (SUS)^a^	73.75 (65–85)
Acceptability of Intervention Measure (AIM)^b^	4 (3.75–4.5)
Intervention Appropriateness Measure (IAM)^b^	4 (4-4.5)
Feasibility of Intervention Measure (FIM)^b^	4 (4-4.5)
Overall, how satisfied are you with this tool?^c^	8 (7–8)
	n(%)
Overall, how did use of the tool impact the length of the visit with your patients?	
Much longer	0 (0.00%)
Somewhat longer	4 (33.33%)
No impact	4 (33.33%)
Somewhat shorter	3 (25.00%)
Much shorter	1 (8.33%)
Overall, how did use of the tool impact the quality of the conversation with the patients?	
Had a positive impact	9 (75.00%)
Had no impact	3 (25.00%)
Had a negative impact	0 (0.00%)
As a result of using this tool my patients spent more time considering the tradeoffs between taking preventive therapy and not taking preventive therapy.	
Strongly agree	4 (33.33%)
Agree	5 (41.67%)
Disagree	3 (25.00%)
Strongly disagree	0 (0.00%)
As a result of using this tool my patients had a better understanding of the evidence about benefits and harms.	
Strongly agree	5 (41.67%)
Agree	7 (58.33%)
Disagree	0 (0.00%)
Strongly disagree	0 (0.00%)
The amount of information provided in the tool was…	
Too much information	1 (8.33%)
Too little information	1 (8.33%)
Just right	10 (83.33%)
Overall, the amount of time it took to review the information in the tool was…	
Too short	0 (0.00%)
Too long	4 (33.33%)
Just right	8 (66.67%)
The tool…	
Favored taking preventive therapy	5 (45.45%)
Favored not taking preventive therapy	0 (0.00%)
Was balanced	6 (54.55%)
How helpful was the tool in helping your patients make a decision about preventive therapy?	
Very helpful	4 (33.33%)
Somewhat helpful	8 (66.67%)
Not helpful	0 (0.00%)
How much did the tool impact your patient’s choice about preventive therapy?	
A great impact	2 (18.18%)
Some impact	7 (63.64%)
A little impact	2 (18.18%)
No impact	0 (0.00%)
Would you use this tool again?	
Yes	9 (90.00%)
No	0 (0.00%)
Unsure	1 (10.00%)
Would you recommend this tool to your colleagues?	
Yes	8 (80.00%)
No	0 (0.00%)
Unsure	2 (20.00%)

IQR: Inter-quartile range^a^The total score of System Usability Scale (SUS) has a range of 0 to 100, where higher numbers refer to higher usability.^b^Scale values of the Acceptability of Intervention Measure (AIM), Intervention Appropriateness Measure (IAM), and Feasibility of Intervention Measure (FIM) range from 1 (completely disagree) to 5 (completely agree).^c^Satisfaction question (Overall, how satisfied are you with this tool? ) was measured on a 9-point Likert scale, where 1 means very unsatisfied and 9 refers to very satisfied.

## Discussion

Our pilot study evaluated the effect of a novel behavior-change tool on decision-making and intent to take preventive therapy among women with premalignant breast lesions at high risk for invasive breast cancer. We examined associations with knowledge about preventive therapy, decisional conflict, decision predisposition, treatment decisions post-physician consultation, and the shared decision-making process. Furthermore, clinicians who participated in the study completed questionnaires and provided feedback on the tool’s acceptability, feasibility and appropriateness. Though the tool was positively received by both patients and clinicians, its use was not associated with differences in participants’ intent to take preventive therapy. Due to the small sample sizes and pilot nature of our study, these findings should be interpreted cautiously and considered exploratory.

Both patients and clinicians perceived the behavior-change intervention as favoring preventive therapy, likely due to the emphasis of the tool on describing the benefits of treatment and magnitude of risk reduction. As a result, the intervention may have influenced decision-making by increasing the participants’ perceived effectiveness of preventive therapy, raising important considerations around balance and equipoise within shared decision-making. Traditional shared decision-making models emphasize a neutral approach in the presentation of options and values clarification to ensure that the decisions made by patients represent their individual preferences [35, 36]. However, for this project, we designed the tool to nudge and encourage preventive therapy given the clinical guidelines that recommend preventive therapy for patients with ALH and LCIS [25]. In situations where there is strong evidence of a net benefit to patients associated with a specific strategy, and the strategy is under-used in the population, there is an ethical justification for using decision aids to encourage a specific choice [26]. In the case of preventive therapy, clinical guidelines endorse its use and there is strong evidence for reduced breast cancer risk, while uptake among eligible patients at a population level is low.

Our study population was highly educated, demonstrated strong health literacy, and was comfortable using digital tools to access health-related information. These characteristics may have contributed to the generally high knowledge levels, especially among participants exposed to the decision tool. Persistent gaps were noted in the knowledge of participants about the frequency of side effects from treatment, accentuating the ongoing challenge of improving preventive therapy uptake. A smaller proportion of women in the post-implementation versus pre-implementation cohort (57% vs. 70%) stated an intent to take preventive therapy. 26% of women in the post-implementation cohort remained unsure about initiating preventive therapy, and 14% decided not to take preventive therapy, often citing concerns about side effects or comorbidities. There is a need for clearer communication around medication side effects and support tools that more effectively address the frequency of treatment-related toxicities and perceived harms.

Medications like tamoxifen tend to be perceived by patients as cancer drugs, and are often associated with unique side effects, which may contribute to participants’ indecision about treatment initiation and their decision to ultimately decline preventive therapy [23, 37]. While the use of these tools to nudge a preferred choice can increase understanding of the risks and benefits of preventive therapy, our findings suggest that the intervention did not achieve its behavioral goal of improving participants’ intent to initiate preventive therapy. Only 38% of intervention participants correctly understood that side effects occur infrequently, possibly indicating that the tool may not have communicated this information effectively. The intervention may also have increased awareness of treatment trade-offs, enabling patients to make well-informed, values-based decisions, even if that meant declining therapy. Regardless, a multifaceted approach that not only provides information to patients, but which also addresses the emotional and psychological barriers to treatment is needed. Some of these concerns, such as fear of side effects may be a significant barrier for patients. Alternative formulations, such as low-dose tamoxifen, which have been reported to have fewer side effects than standard-dose tamoxifen and aromatase inhibitors are now available for use [38, 39]. Future studies should explore whether these low dose formulations improve the uptake of preventive therapy, particularly among patients who might be hesitant to initiate treatment due to side effect concerns.

Decisional conflict reflects the discomfort and internal struggle experienced when people are faced with complex choices. In a pre-post study conducted by Stacey et al., the use of a decision aid alone and in combination with counselling about preventive therapy for breast cancer reported a significant decrease in the number of participants undecided about preventive therapy, suggesting a decrease in decisional conflict [24]. Similar findings have been reported in studies using decision tools based on the Ottawa decision support framework [40–43]. In our study, overall decisional conflict was low in both groups, and median scores on the decisional conflict subscales were generally similar. The high scores on the shared decision-making process in both groups reflect the strong value participants placed on clinician engagement, regardless of intervention exposure.

Our findings also emphasize the importance of providing clinicians with tools that facilitate discussions about preventive therapy. The positive feedback from clinicians about the acceptability, feasibility, and appropriateness of the tool indicates their readiness to integrate it into clinical practice. Addressing this need among clinicians could support more consistent and meaningful conversations about preventive therapy in clinical encounters. To maximize the impact, clinicians must also receive adequate training and support to confidently use these tools in discussions with patients.

Our pilot study is not without limitations. Participants were recruited from a single institution and were predominantly well-educated, non-Hispanic White women which limits the generalizability of our findings. There was a delay in conducting the post-implementation phase and recruitment was slowed by the COVID pandemic. Furthermore, our sample size was small and hence, may not have sufficient power to detect statistically significant differences between groups. Since participants were not randomly assigned to groups, the study may be prone to selection bias. However, the sociodemographic characteristics were similar between cohorts. No baseline data were assessed, hence the differences between groups may be due to confounding variables that were not controlled for. In addition, we evaluated participants’ intent to take preventive therapy but not actual initiation. Therefore, it is unknown whether patients followed through with the decision or changed their minds over time. Nonetheless, our findings highlight the high value that patients place on collaborative discussions with their clinicians about preventive care options, and the usefulness and acceptability of a novel strategy to improve knowledge about preventive therapy and reduce decision uncertainty. Future research should incorporate objective measures of therapy initiation and continuation, focus on tools that directly address patient concerns about side effect frequency and management, incorporate lower-dose therapy options and bridge the complex gap between knowledge, shared decision-making, and treatment uptake.

## Electronic supplementary material

Below is the link to the electronic supplementary material.

Supplementary material 1

## Data Availability

The data that support the findings of this study are available on reasonable request from the corresponding author. The data are not publicly available due to privacy or ethical restrictions.

## Declarations

### Running Head

Behavior-Change Intervention for High-Risk Women.

### Prior Presentation

SABCS 2024 Annual Meeting in San Antonio, TX, December 10–14, 2024.
