# Supplementary material for: A pilot study of a behavior-change intervention for preventive therapy among women at high risk for invasive breast cancer
Source: BMC Med Inform Decis Mak. 2026 Apr 18;26:193. doi: 10.1186/s12911-026-03510-4 (PMC13220379; doi:10.1186/s12911-026-03510-4)
Supplement: Supplementary file 1 — Supplementary material 1 [file 12911_2026_3510_MOESM1_ESM.docx]

| Supplementary Table 1. Participants' Knowledge about Breast Cancer Preventive Therapy (N=48) | | | |
| --- | --- | --- | --- |
|  | **Pre-implementation (n=27)** | **Implementation (n=21)** | **p-value** |
| **Total knowledge score, median (IQR)*** | 0.83 (0.67-0.83) | 0.83 (0.83-1.00) | **0.016** |
|  |  |  |  |
| **How do women with lobular carcinoma in situ (LCIS) or atypical hyperplasia (AH) compared to other women?** |  |  | 0.50 |
| Incorrect/Unsure | 2 (7.41%) | 0 (0.00%) |  |
| Correct | 25 (92.59%) | 21 (100.00%) |  |
|  |  |  |  |
| **Taking preventive therapy can lower a woman’s risk of developing breast cancer by:** |  |  | 1.00 |
| Incorrect/Unsure | 2 (7.41%) | 1 (4.76%) |  |
| Correct | 25 (92.59%) | 20 (95.24%) |  |
|  |  |  |  |
| **Preventive therapy can cause side effects such as hot flashes and vaginal symptoms.** |  |  | 0.25 |
| Incorrect/Unsure | 3 (11.11%) | 0 (0.00%) |  |
| Correct | 24 (88.89%) | 21 (100.00%) |  |
|  |  |  |  |
| **Side effects from preventive therapy happen infrequently:** |  |  | 0.34 |
| Incorrect/Unsure | 21 (77.78%) | 13 (61.90%) |  |
| Correct | 6 (22.22%) | 8 (38.10%) |  |
|  |  |  |  |
| **Preventive therapy has greater effectiveness when you take the drug for 5 years?** |  |  | 0.21 |
| Incorrect/Unsure | 5 (18.52%) | 1 (4.76%) |  |
| Correct | 22 (81.48%) | 20 (95.24%) |  |
|  |  |  |  |
| **Compared to other activities that can lower the risk of health problems (such as taking cholesterol-reducing medication to prevent a heart attack or getting a flu shot to prevent the flu), preventive therapy is:** |  |  | 0.083 |
| Incorrect/Unsure | 9 (33.33%) | 2 (9.52%) |  |
| Correct | 18 (66.67%) | 19 (90.48%) |  |
| IQR: Inter-quartile range  P-value for total knowledge score is based on Wilcoxon Rank Sum test. For all other variables, p-value is based on Exact Fischer test  *Total knowledge score is the percentage correct | | | |
